# Screening method and metabolic analysis of plant anti-aging microorganisms via ammonia-induced senescence in the duckweed *Wolffia microscopica*

**DOI:** 10.3389/fpls.2024.1480588

**Published:** 2024-11-13

**Authors:** Deguan Tan, Lili Fu, Ying Yu, Xuepiao Sun, Jiaming Zhang

**Affiliations:** ^1^ National Key Laboratory for Tropical Crop Breeding, Institute of Tropical Bioscience and Biotechnology, Sanya Research Institute of Chinese Academy of Tropical Agricultural Sciences, Haikou, China; ^2^ Hainan Key Laboratory of Microbiological Resources, Hainan Institute for Tropical Agricultural Resources, Chinese Academy of Tropical Agricultural Sciences, Haikou, China

**Keywords:** duckweeds, anti-aging microorganism, stay-green, screening model, endophytes, comparative metabolome

## Abstract

Ammonium is the preferred N nutrition over nitrate for some plant species, but it is toxic to many other plant species and induces senescence at high concentrations. The duckweed *Wolffia microscopica* (Griff.) Kurz is the smallest and fast-growing angiosperm. It is highly sensitive to ammonium and has a short lifespan on media containing 0.5 mM or higher ammonia. This feature makes it a potential model plant to screen for anti-aging microorganisms. By co-culturing *W. microscopica* with endophytic microorgainisms isolated from rubber tree, we screened out an *Aspergillus sclerotiorum* strain ITBB2-31 that significantly increased the lifespan and the biomass of *W. microscopica*. Interestingly, both filter-sterilized and autoclaved exudates of ITBB2-31 increased the lifespan of *W. microscopica* cultures from 1 month to at least 7 months. Meanwhile, the exudates also showed strong anti-aging effects on cassava and the rubber tree leaves and increased chlorophyll contents by 50% - 350%. However, high contents of filter-sterilized exudates inhibited the growth of *W. microscopica* while extending its lifespan, indicating that there were heat-sensitive growth-inhibiting agents in the exudates as well. Comparative metabolome analysis of the filter-sterilized and autoclaved exudates revealed multiple heat-stable anti-aging and heat-sensitive growth-inhibiting compounds. Our results suggest that *W. microscopica* can be served as a rapid and efficient model plant to screen for plant anti-aging microorganisms.

## 1 Introduction

Leaf aging is one of the important factors that influence crop yields. The stay-green characters of leaves extend the duration of active photosynthesis and are positively correlated with yields and stress tolerance in many crops, including maize, wheat, rice, soybean, sorghum, oats, and cassava (Rawson et al., 1983; Sasaki and Ishii, 1992; Bekavac et al., 2002; Joshi et al., 2007; Kusaba et al., 2013; Thomas and Ougham, 2014). The stay-green genotypes of maize, for example, do not lose the green color of their leaves until physiological maturity, while the non-stay-green genotypes start losing the green color of their leaves approximately 30 days after flowering (Bekavac et al., 2002). Stay-green alleles enhance grain yield in sorghum under drought by modifying canopy development and water uptake patterns (Borrell et al., 2014). Exogenous application of cytokinins on leaves of winter wheat improved stay-green characteristics and thus increased grain yield in heat stress (Yang et al., 2016). Therefore, stay-green traits have been used in breeding programs of many grain crops. Through intensive selection, varieties with both higher yields and longer duration of greenness in a range of grain crops have been commercialized (Thomas and Ougham, 2014). Stay-green is also important to root crops. For example, cassava plant ideally has a leaf life of 15–20 weeks and an optimal leaf area index (LAI) of 4 to 6 according to a simulation model for substantially increased cassava yields with longer leaf life (Cock et al., 1979). In most cultivars, however, leaf life is much shorter, and replacement of leaves requires energy input and creates a competitive sink for photosynthetic assimilates, thus reducing root yields. Introduction of a senescence-inducible isopentyl transferase gene into cassava extended leaf greenness, and thus increased the yield (Zhang et al., 2010). The importance of stay-green in agriculture and the relevant molecular mechanisms that regulate stay-green traits have been well reviewed (Kusaba et al., 2013; Thomas and Ougham, 2014; Abdelrahman et al., 2017).

The stay-green characters are usually obtained by natural mutation and/or artificial engineering. An alternative way may be the application of beneficial microorganisms to plants. Beneficial microorganisms have been isolated from many plants as endophytes. The interactions between hosts and endophytes involve flows of various compounds, of which some are highly toxic to the hosts (Powell and Petroski, 1992), however, in many cases, the endophytes are beneficial to hosts by increasing abiotic and biotic stress resistances, and thus improve the adaptation of the plants (Schardl et al., 2004). Some grass species from coastal and geothermal habitats require symbiotic fungal endophytes for salt and heat tolerance (Rodriguez and Redman, 2008). The ability of symbiotic microorganisms to confer stress tolerance to plants may provide a novel strategy to cope with the problems in modern agriculture. In this paper, we report a rapid and efficient method for screening of anti-aging microorganisms, based on an early aging duckweed species *Wolffia microscopica* using *in vitro* culture system.

Duckweeds belong to a globally distributed family Lemnaceae. They are free floating monocots and are currently classified into 5 genera and 37 species (Landolt, 1986; Appenroth et al., 2013). Duckweed clones are collected from all over the world and are maintained at Duckweed Stock Collection Centers situated in Zurich, Switzerland; Jena, Germany; Rutgers, New Brunswick, USA; and Chengdu and Haikou, China. The most accepted maintenance media is the modified Hoagland medium (Hoagland and Arnon, 1950), N, E, and SH media supplemented with 1% sucrose and solidified with 0.6% agar (Appenroth, 2015; Sree and Appenroth, 2016). While most clones can be stored for three months before subculture, some clones should be subcultured more often, such as *W. microscopica*.

**Figure 1:**
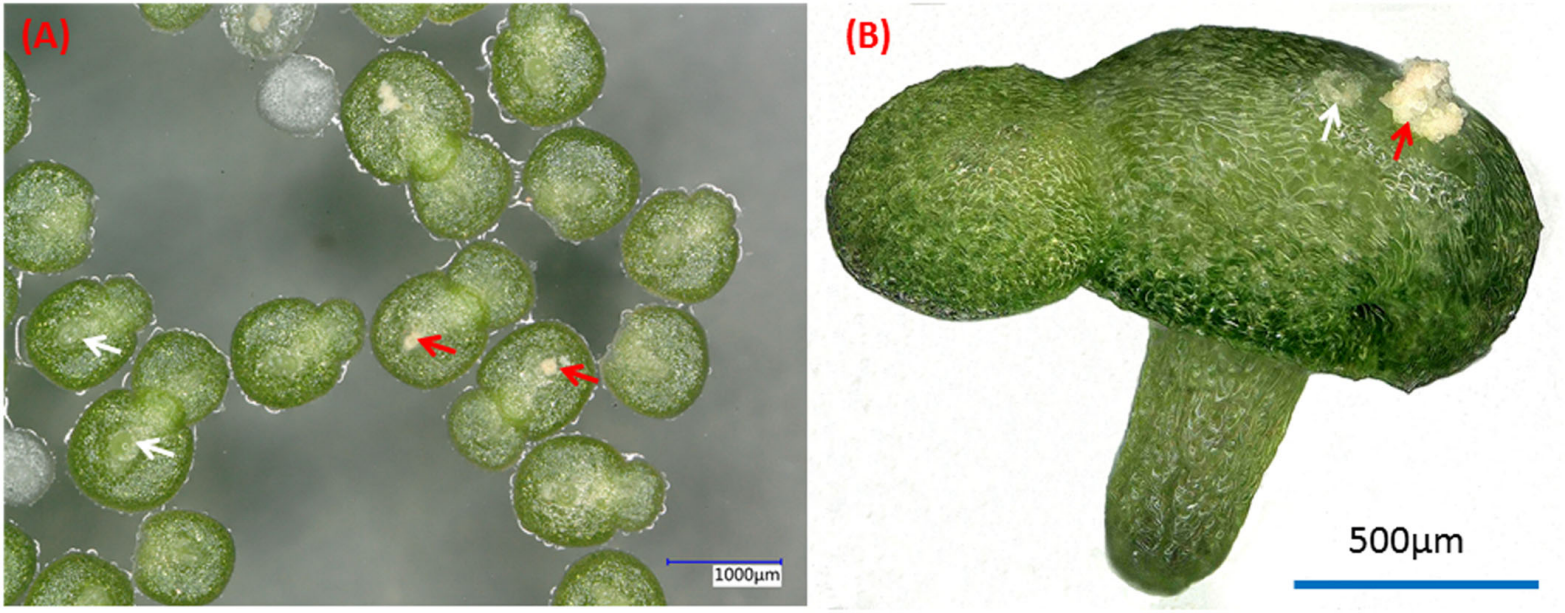
Dorsal **(A)** and side **(B)** views of typical two-fronds plant of *W. microscopica* 2001. White arrows indicate the pistils and red arrows indicate the stamens.

*W. microscopica* is one of the fast growing duckweed species with a biomass doubling time of only one day (Ziegler et al., 2015; Appenroth et al., 2017). Its plant body is simplified into a tiny frond with a size of only around 1 mm (**Figure 1**). However, its axenic stock cultures senesce more rapidly, resulting in a shorter maintenance cycle compared to the other species in the duckweed family, thus requiring to be subcultured more often. This species was once lost completely from all duckweed collections in the year 2009; however, it was rediscovered in 2013 (Sree and Appenroth, 2014).

The rubber tree (*Hevea brasiliensis*) is widely cultivated in tropical areas (Heng and Joo, 2017) where there exist rich environmental microorganisms, and its trunk bark is regularly cut for harvesting latex, thus making it more susceptible to external microbial invasion. In addition, the rubber tree mainly reproduces through bud grafting (Heng and Joo, 2017), which keeps the spread of endophytes among its offsprings. These make the rubber tree abundant in endophytic microorganisms. Many endophytic fungal strains from the rubber tree have been discovered, among which *Ascomycota* species accounts for 97%, and the sapwood has a greater diversity of endophytes than the leaves (Gazis and Chaverri, 2010). Our research team has also isolated many endophytic fungi from the rubber tree, and found that some strains can remarkably inhibit the growth of the pathogenic fungus *Colletotrichum gloeosporioides* Penz. Sace and *Fusarium oxysporum* Cubense (Zheng et al., 2009a), and another strain called ITBB2-1 exhibited high salt tolerance (Zheng et al., 2009c). These findings indicate that endophytic fungi of the rubber tree have some unique biological functions.

In this study, we found that *W. microscopica* is sensitive to ammonia-induced senescence. The early-aging phenomena can be served as a reporter to screen for anti-aging microorganisms by co-culturing it with candidate microorganisms or by supplementation of the fermented medium of microorganisms into the culture medium of the plant.

## 2 Materials and methods

### 2.1 Plant materials and culture media

The axenic *Wolffia microscopica* (Griff.) Kurz clones 2001 and 2008 were provided by Prof. Klaus-J. Appenroth at University of Jena, Germany and Dr. K. Sowjanya Sree at Central University of Kerala, India. The clones are maintained at the Institute of Tropical Bioscience and Biotechnology (ITBB), Chinese Academy of Tropical Agricultural Sciences (CATAS). The basic subculture medium was a modified Hoagland medium (MH) originally designated by Hoagland and Arnon (1950) supplemented with 29 mM sucrose and 6 g L^-1^ agar (Biotechnology grade, Beijing Solarbiao Science & Technology co., Ltd, Beijing, China) for solidification. The components of the MH medium was previously described (Fu et al., 2017). Other media that were tested in this research include N (Appenroth et al., 1996), E (Cleland and Briggs, 1967), and SH (Schenk and Hildebrandt, 1972) media (**Supplementary Table S1**). The media were autoclaved at 121°C for 20 min.

### 2.2 Stay-green verification of the stock cultures of *W. microscopia*

Ten plants of *W. microscopia* clones 2001 and 2008, respectively, were inoculated in the centre of each 6-cm plate containing MH medium solidified with 6 g L^-1^ agar at 5 d intervals, 6 plates were inoculated each time for each clone. The plates were incubated in a growth chamber (BIC-250, Boxun Instrument, Shanghai, China) with 16 h photoperiod at 100 μmol·m^-2^·s^-1^ photosynthetic active radiation (PAR) and 25 ± 1°C. The status of the plants was examined every other day until two months, and then fronds were harvested and weighed. Three parallel experiments were performed.

### 2.3 Screening of anti-aging fungal strains by coculturing *W. microscopica* with candidate fungi

The endophytic fungal strains were previously isolated from the rubber tree (Zheng et al., 2009a). The fungi were maintained on Potato Dextrose and Agar (PDA) medium at room temperature. Ten fronds of *W. microscopia* clone 2001 were inoculated in the centre of each 6-cm plate containing MH medium. Fungal spores and/or mycelia were co-cultured on the edge of the same plate. The plates that were only inoculated with *W. microscopia* were used as control. All plates were incubated in the growth chamber (BIC-250, Boxun, Shanghai) with 16 h photoperiod at 100 μmol·m^-2^·s^-1^ photosynthetic active radiation (PAR) and 25 ± 1°C for 30 d. The status of the plants was examined every other day.

### 2.4 Temperature-dependence of growth-promoting effect of fungal strain ITBB2-31

The growth-promoting experiments of *Aspergillus sclerotiorum* strain ITBB2-31 was carried out as described above. The plates were incubated in growth chambers (BIC-250, Boxun, Shanghai) with 16 h photoperiod at 100 μmol·m^-2^·s^-1^ photosynthetic active radiation (PAR) for 30 d at 20°C, 25°C, and 30°C, respectively. The fronds in each plate was wrapped in tinfoil and dried at 105°C overnight and weighed. The significance of weight differences was analyzed using IBM SPSS Statistics Version 24 (IBM Corporation, New York, USA).

### 2.5 Anti-aging effects of ITBB2-31 fermented broth on *W. microscopica*

The fungal strain ITBB2-31 was inoculated in MH liquid medium supplemented with 29 mM sucrose and incubated at 28°C in the dark for 10 d. The fermented broth was filtered with 50 µm nylon membrane to remove the mycelium. The filtrate was then passed through filter paper, and filter-sterilized with 0.22 µm filter units (Millipore, Bedford, USA), and then added into the sterilized MH medium with concentrations of 10%, 20%, 30%, and 50% (v/v), respectively. To test whether the functional element in the exudate was temperature resistant, the filtrate was added into MH medium with above concentrations and autoclaved at 121°C for 20 min. The autoclaved media were poured into 6-cm plates. *W. microscopia* clones 2001 and 2008 were inoculated in the centre of the plates and incubated in the growth-chamber with 16 h photoperiod at 100 μmol·m^-2^·s^-1^ photosynthetic active radiation (PAR) and 25°C for one month. The plants in each plate were separately wrapped in tinfoil and dried at 105°C overnight and weighed. The significance of weight differences between treatments was analyzed using IBM SPSS Statistics Version 24.

### 2.6 Anti-aging effect of ITBB2-31 on the rubber tree and cassava leaves

The anti-aging effect of ITBB2-31 was tested using the leaves of test-tube plants of rubber tree and cassava following the method described by Zhang et al (Zhang et al., 2010). Fully expanded, green, and healthy leaves collected from rubber tree plants grown in the greenhouse were also used. The leaves were put in 15-cm petri-dishes on filter paper wetted with MH medium supplemented by 0%, 10%, 20%, and 30% fermented MH broth of ITBB2-31. Dark-induced senescence was performed by incubating the leaves in the dark at 25°C for two weeks. The leaf aging status was recorded by photography every day. To quantify the anti-senescence effect, leaf samples were homogenized in liquid nitrogen and dissolved in 100% (v/v) acetone as described in a method (Lichtenthaler, 2010). The samples were incubated at room temperature for 30 min and centrifuged to remove cell debris. Chlorophyll *a* and *b* content of the supernatants was determined by spectrophotometer and calculated according to the equation as previously described (Lichtenthaler, 1987). The significance of the differences between treatments was tested by one-way ANOVA and LSD test using IBM SPSS Statistics Version 24.0 (IBM Corporation, New York, USA).

### 2.7 DNA extraction and polymerase chain reaction and phylogenetic analysis

Genomic DNA of fungal strain ITBB2-31 was isolated using a fungal DNA isolation kit (Tiangen Biotech, Beijing, China). The internal transcribed spacer (ITS) sequence was amplified as previously described (Zheng et al., 2009c) with two primers HNP76 (5′-TCCGTAGGTGAACCTGCGG-3′) and HNP77 (5′-TCCTCCGCTTATTGATATGC-3′). The amplified fragment was sequenced on both strands at BGI (Beijing Genomic Institute), Shenzhen, China. For phylogenetic analysis, reference sequences were downloaded from GenBank. The sequences were aligned with MacVector 15.0.2, the unaligned 5′ and 3′ sequences were removed. Phylogenetic tree was generated using Mega7 (Kumar et al., 2016). The evolutionary history was inferred by using the Maximum Likelihood method based on the Tamura-Nei model (Tamura and Nei, 1993). The tree with the highest log likelihood is shown. The tree is drawn to scale, with branch lengths measured in the number of substitutions per site. All positions containing gaps and missing data were eliminated, and there was a total of 453 positions in the final dataset.

### 2.8 LC-ESI-MS/MS analysis of the fermented media

Both filter-sterilized and autoclaved fermented media of ITBB2-31 were respectively analyzed using liquid chromatography electrospray ionization tandem mass spectrometry (LC-ESI-MS/MS). For liquid chromatograph (LC), an Agilent 1290 (Agilent Technologies, CA) with a Waters column ACQUITY UPLCBEH Amide (1.8 μm, 2.1 mm × 100 mm) was used. The column temperature was set to 35°C, and 2 μL of sample was injected. The elution buffers for positive ion model were 0.1% formic acid in water (buffer A) and 0.1% formic acid in acetonitrile (buffer B); 95% of buffer A was supplied for the first minute of run, then the profile of buffer B was increased gradually until reaching 95% of the elution buffer at 16 minutes, and reduced to 5% at 18 minutes. For negative ion model, 2 mM ammonia acetate (buffer A) and acetonitrile (buffer B) were used, following the same elution schedule as above. The flow rate was 0.4 mL/min. For mass spectrometry, an Agilent 6545QTOF with control software LC/MS Data Acquisition, Version B.08.00 was used. The ion source temperature was 320°C. The nitrogen gas flow and sheath gas flow were 8 L/min and 12 L/min, respectively. Sheath gas temperature was 350°C. The auxiliary pressure was set to 3500 V and 4000 V for negative and positive models, respectively. Mass spectra of the parent ions and the subsequent fragmented ions were scanned over the range of 50–1100 m/z. The data was analyzed using software MSDIAL (version 2.54) and the identities of the metabolites were determined by searching against the in-built MS/MS reference libraries including metlin, MassBank, MoNA, and HMDB. Relative abundance of a compound was calculated using the equation: the area of the compound/the total area of all compounds × the number of compounds.

## 3 Results

### 3.1 Ammonium-induced senescence observed by *in vitro* culture of *W. microscopica*

**Figure 2:**
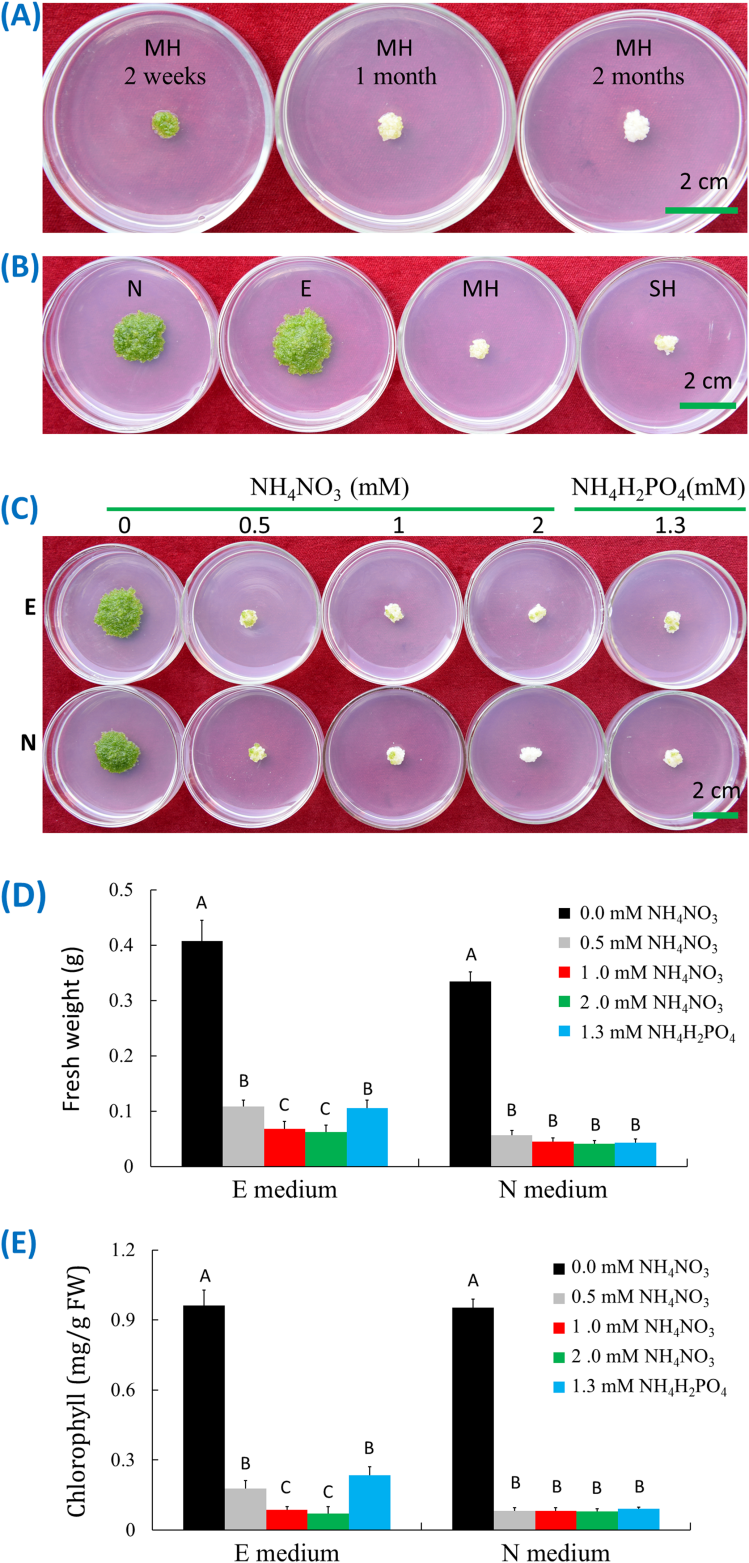
Ammonia-induced senescence of *W. microscopica* 2001. **(A)** Growth status on MH medium at different time points as indicated on the plates; **(B)** Growth status on different culture media as indicated on the plates; **(C)** Growth status on E and N media supplemented with different types and concentrations of ammonia salts; **(D)** Fresh weight analysis of plant colonies in **(C)**; **(E)** Chlorophyll content analysis of plant colonies in **(C)**. The culture temperature was 25°C and the culture time was four weeks if not indicated. The significance of data was analyzed with one-way ANOVA and LSD test. Different letters on columns indicate significance at 1% level.

The MH medium is one of the commonly used media to retain the axenic stock cultures of duckweed species. However, one species, *W. microscopica* did not perform well on MH medium. This species grew at the beginning, and reached a colony size of approximately 0.8-1.3 cm in diameter in two weeks, and then approached senescence (**Figure 2A**). Most colonies died within one month with a few exceptions in some colonies, and all plants died without exceptions in two months (**Figure 2A**). Therefore, the lifespan of *in vitro* cultures of *W. microscopica* on MH medium was only one month. In contrast, the other species in the duckweed family can be subcultured at three-month intervals on MH medium. This is probably the reason why *W. microscopica* was easy lost from the duckweed stock collections (Sree and Appenroth, 2014).

To investigate the reason of early senescence in *W. microscopica* stock cultures, four commonly used media including MH, SH, E, and N media were used to culture *W. microscopica* clone 2001. The plants grew the fastest on E medium, followed by N medium, and slowest on SH and MH media. In the meantime, the plants on MH and SH media began senescence rapidly and mostly turned pale white within three weeks and could not resume growth on fresh medium, while the plants on E and N media were still prosperous (**Figure 2B**). By comparing the components in the four media, we found that both MH and SH media contained ammonia nitrogen (**Supplementary Table S1**), which were 1 mM NH_4_NO_3_ and 1.3 mM (NH_4_)H_2_PO_4_ in MH and SH media, respectively, while E and N media contained only nitrate.

Ammonia-induced senescence has been reported in both mammals and plants (Lin et al., 2002; Gorg et al., 2015). To investigate whether the early senescence of *W. microscopica* on MH and SH media was caused by ammonia-induced senescence, *W. microscopica* clones 2001 and 2008 were cultured on E and N media supplemented with different concentrations of ammonium ion. Results indicated that supplementation of 0.5 mM NH_4_^+^ in both E and N media was enough to drive *W. microscopica* to die within four weeks (**Figure 2C**), the higher the NH_4_^+^ concentration, the quicker for *W. microscopica* to die. The fresh weights were reduced by 73% - 85% in E medium and 83% - 88% in N medium depending on the concentration of ammonium salts (**Figure 2D**). The chlorophyll contents were reduced by 81% - 92% in the E and N media after addition of ammonium salts (**Figure 2E**). These results indicated that the early senescence of *W. microscopica* was induced by ammonia ion.

### 3.2 Screening of anti-aging microorganisms

**Figure 3:**
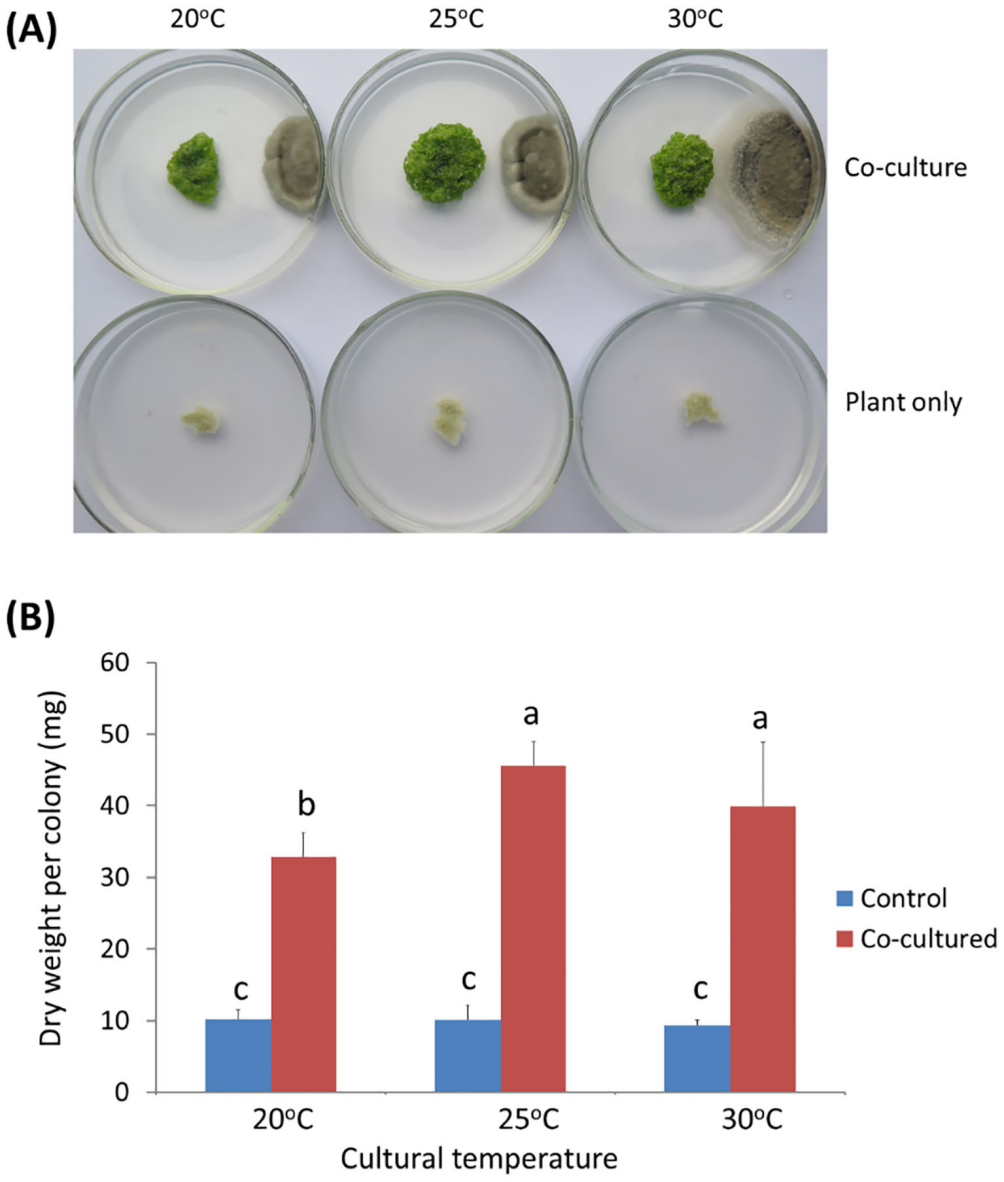
Anti-aging effects of endophytic fungal strain ITBB2-31 by co-culture with *W. micoscopica* 2001. **(A)** Representative plates of co-culture and control after incubation for one month under different temperatures; **(B)** Statistic analysis of the dry weights of *W. micorscopica* colonies. The significance of differences was tested by one-way ANOVA and LSD test. Different letters above columns indicate significant differences at 5% level.

Twenty-five endophytic microbial strains isolated from rubber tree (Zheng et al., 2009a) were tested for their anti-aging effect on *W. microscopica*. One strain designated as ITBB2-31 showed strong anti-aging activity. When *W. microscopia* clones was co-cultured with ITBB2-31, its growth was significantly promoted, and the co-cultured plant colonies grew to a larger diameter and kept growing after one month of inoculation at all tested temperatures (**Figure 3A**). In contrast, the control colonies that were not co-cultured had stopped growth in approximately two weeks and died in one month (**Figure 3A**). The dry weight of co-cultured colonies was 3.5-4.5 times the weights of the controls depending on the culture temperatures, and the highest dry weight (45.6 mg) was obtained at 25°C (**Figure 3B**).

### 3.3 Molecular identification of ITBB2-31

The internal transcribed spacer sequence (ITS) of strain ITBB2-31 was amplified by PCR method. The amplified product was 609 bp. BLAST searches against the NCBI database resulted in best hit to *Aspergillus sclerotiorum* Huber strain ATCC16892, which is the type strain of *A. sclerotiorum* and was isolated from fruit of apple (*Malus sylvestris*) by G.A. Huber in Oregon, USA (Huber, 1933). Phylogenetic analysis indicated that ITBB2-31 belonged to the yellow aspergilli, the *Aspergillus* section *Circumdati* (Visagie et al., 2014), and formed a clade with *A. sclerotiorum* strains and *A. subramanianii*, *A. bridgeri*, *A. persii*, *A. salwaensis* and *A. roseoglobulosus* strains with 98% bootstrap support (**Supplementary Figure S1**). Pairwise alignments indicated that the ITS sequence of strain ITBB2-31 had 100% identities with strains ATCC16892, NW3, NRRL35202, NRRL415, NRRL35024, DTO129-F5, and CCF3434; and 99.6% - 99.8% identities compared to *A. scelrotiorum* strain ANDEF08, *A. subramanianii* strains DTO129G4, NRRL6161 and DTO245E4, *A. bridgeri* strain NRRL1300, and *A. persii* strain CBS112795. The next closely related strains in the same clade were *A. salwaensis* DTO297B3T with 99.2% identities and *A. roseoglobulosus* strain CBS112800 with 97.1% identities. The sequence identities compared to the strains in other clades were less than 92.3%. Taken together, ITBB2-31 is an *A. scelrotiorum* strain.

### 3.4 The fermented broth of ITBB2-31 presents anti-aging activity for *W. microscopia*

**Figure 4:**
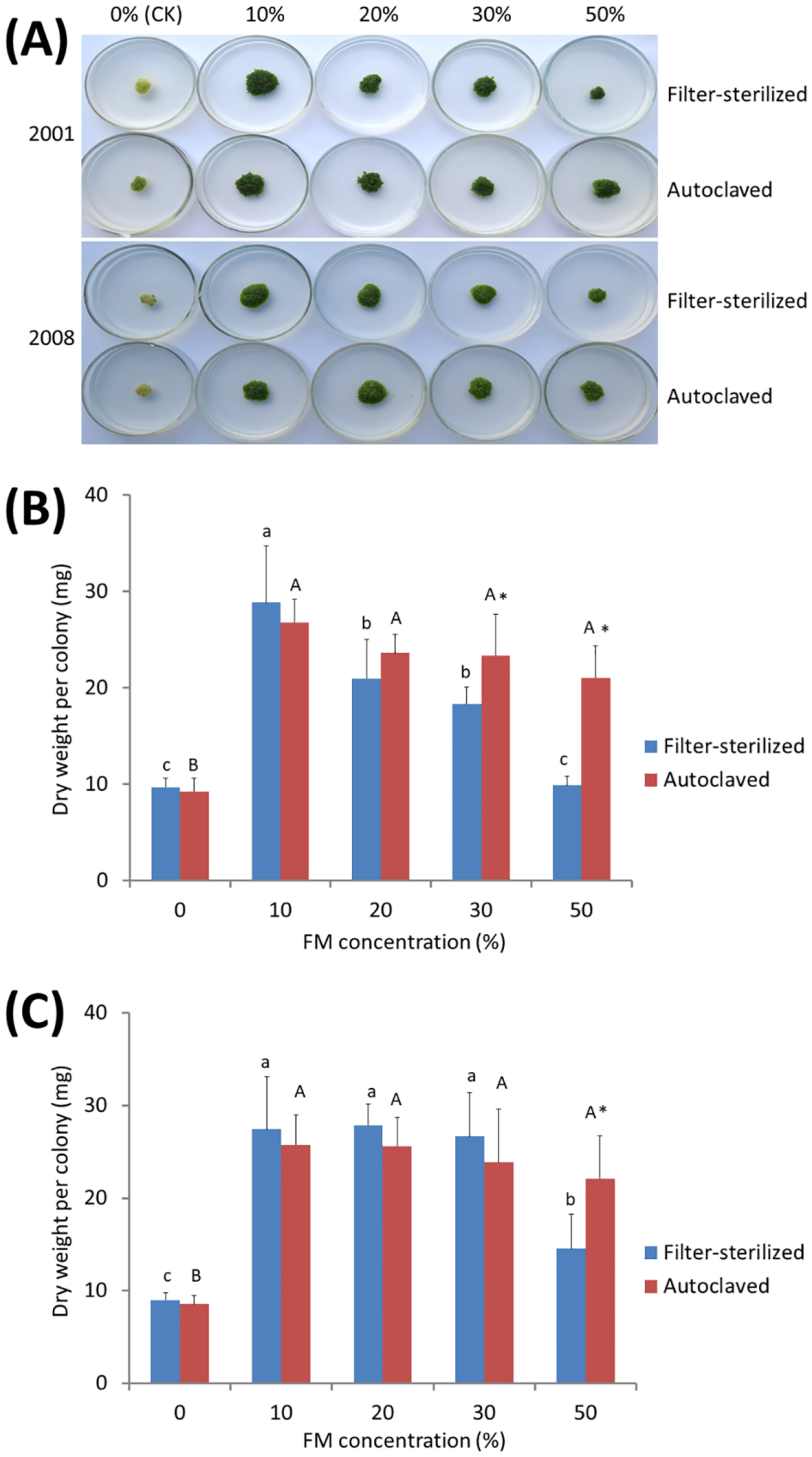
Anti-aging effects of the exudates of fungal strain ITBB2-31 on *W. microscopica* clones 2001 and 2008. Both autoclaved and filter-sterilized fermented MH broth (FM) were supplemented to the MH medium with different proportions as indicated in the figure. **(A)** growth status of the two strains after one month of culture at 25°C; **(B, C)** statistical analysis of dry weights of clones 2001 **(B)** and 2008 **(C)**. The significance of differences among the proportions of filter-sterilized (lower case letters) and the autoclaved (Upper case letters) FM was tested by one-way ANOVA and LSD methods. Different letters above columns indicate significant differences at 5% level of significance. The differences between the filter-sterilized and the autoclaved FM at the same proportion was tested with Independent-Samples T-test, and (*) above the columns indicates significance at 5% level.

It is obvious that the anti-aging and growth-promoting activity of ITBB2-31 did not require physical contact between the plants and fungal colonies (**Figure 3A**), because the fungus and plants were inoculated far away, and in most cases the fungus and plants remained separated in one month. Therefore, there must be some components secreted into the medium or some volatiles released to the air by the fungus. We tested the medium in this research, either autoclaved or filter-sterilized fermented MH medium (FM hereafter) was added to the fresh MH medium in different proportions before inoculation of the plants. Results indicated that both autoclaved and filter-sterilized FM prevented senescence and promoted the growth of *W. microscopia*. The plants of both clones 2001 and 2008 kept alive and continued to grow at all tested FM concentrations (**Figure 4A**). In contrast, the plants of both clones that grew on the control medium died in one month (**Figure 4A**). Therefore, the anti-aging factor was thermo-stable and was secreted into the culture medium.

Besides the anti-aging and growth-promoting effect, supplementation with 30% or higher concentrations of the filter-sterilized FM resulted in lower growth-rate of *W. microscopia* as compared to 10% filter-sterilized FM, although the plants stayed green (**Figure 4A**). This inhibition effect was significant when the proportion of the filter-sterilized FM was increased to 50% (**Figures 4B, C**). However, the autoclaved FM present very weak inhibition effect and the decrease of dry weight at high concentrations was not statistically significant (**Figures 4B, C**), and the dry weights of plant colonies grown on 50% of autoclaved FM were significantly higher than those grown on 50% filter-sterilized FM for both clones 2001 and 2008 (**Figures 4B, C**). These results indicated that the components that inhibited plant growth were sensitive to high temperatures (e.g. 121°C).

**Figure 5:**
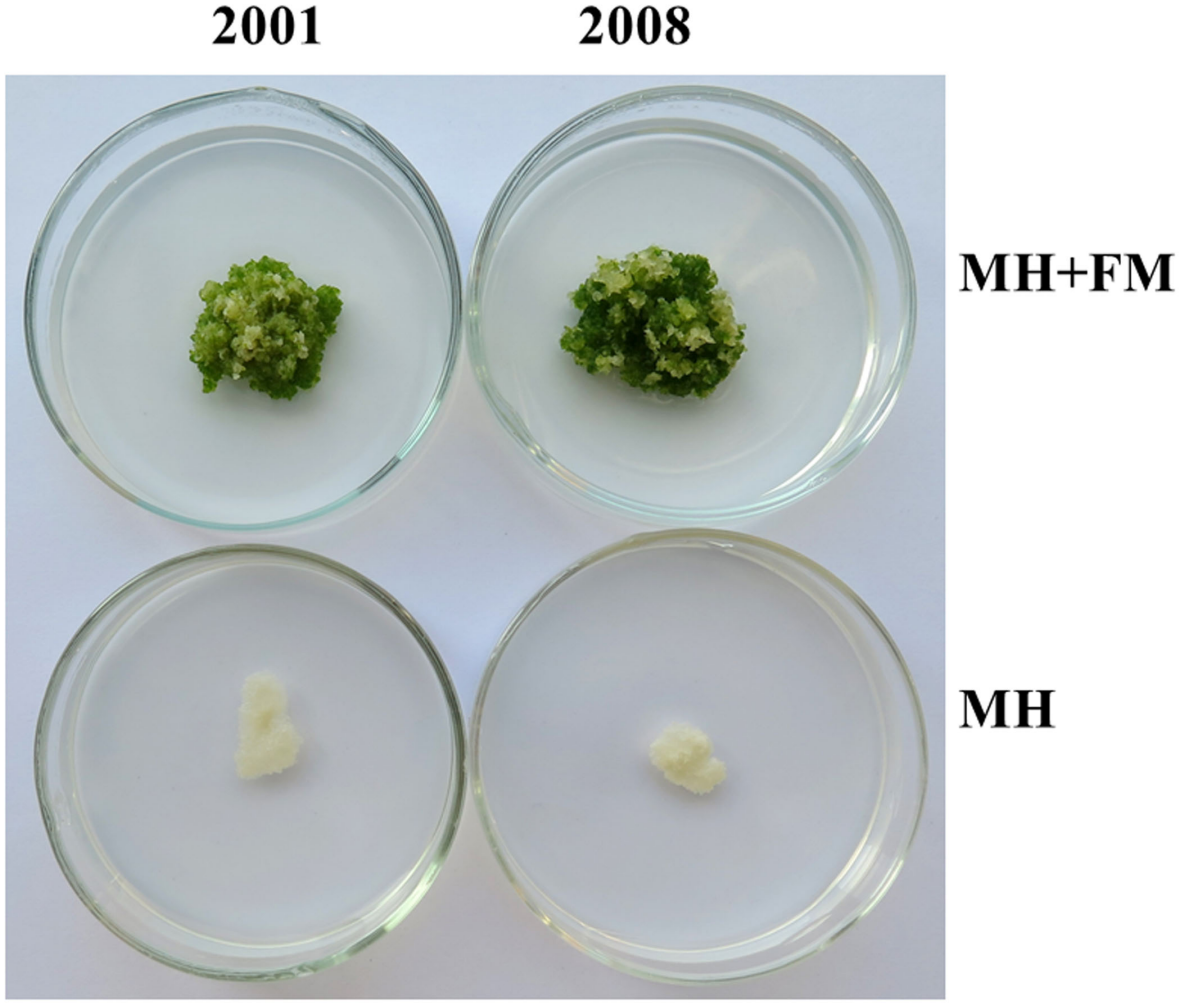
Long lasting anti-aging effects of the exudates of fungal strain ITBB2-31 on *W. microscopica* clones 2001 and 2008. Representative plates of *M. microscopica* clones 2001 and 2008 incubated on MH medium supplemented with 10% FM at 25°C for seven months. The medium without addition of FM was used as control.

Moreover, the exudates of ITBB2-31 presented anti-aging activity to *W. microscopica* for many months. *W. microscopica* clones 2001 and 2008 were successfully retained on MH medium supplemented with 10% autoclaved FM for more than 7 months at 25°C without subculture (**Figure 5**).

### 3.5 Anti-aging activity of ITBB2-31 against dark-induced senescence of the rubber tree and cassava leaves

**Figure 6:**
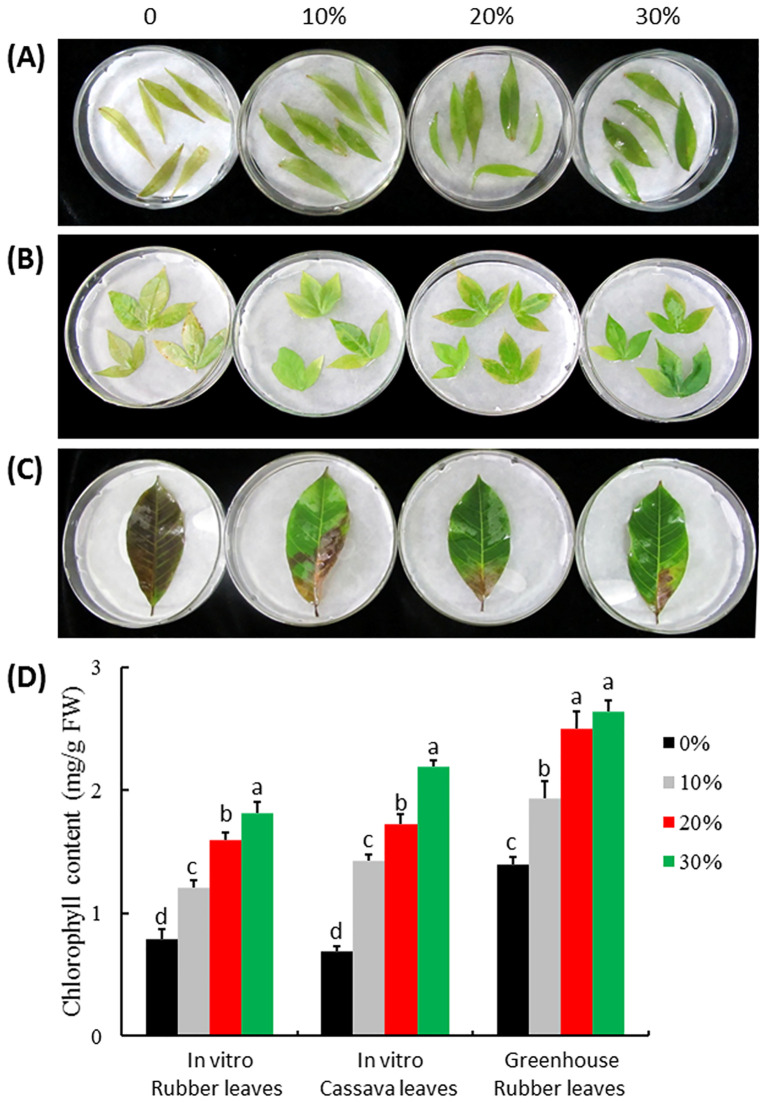
Anti-aging effect of ITBB2-31 on detached leaves of rubber tree and cassava. Leaves were detached from *in vitro* plantlets of rubber tree variety Reyan7-33-97 **(A)** and cassava variety SC5 **(B)** and from field plants of rubber tree Reyan7-33-97 **(C)**; The leaves were incubated on different concentrations of autoclaved FM at 25°C for 15 days in the dark; **(D)** Statistical analysis of chlorophyll contents in the leaves. Different letters above columns indicate significant difference at 5% significance level.

The FM of fungal strain ITBB2-31 presented strong anti-aging effects to detached leaves of *in vitro* plantlets of the rubber tree (**Figure 6A**) and cassava (**Figure 6B**), and the rubber tree leaves collected from the field (**Figure 6C**). Obvious senescence was shown on the control leaves of *in vitro* rubber tree and cassava after 15 days of dark treatment, and the chlorophyll contents of the leaves were only 0.79 mg/g and 0.70 mg/g FW for the rubber tree and cassava, respectively. Adding 10% FM significantly reduced senescence and increased chlorophyll contents by 53.6% and 106% for *in vitro* rubber tree and cassava leaves, respectively. The higher the concentration of FM, the higher the anti-aging effect (**Figure 6D**).

The anti-aging effect of ITBB2-31 on the rubber tree leaves collected from the field was also significant. The control leaves turned brown with chlorophyll content of 1.40 mg/g FW after 15 days of dark treatment. The leaves treated with 10% FM of ITBB2-31 had 50% green area, and the total chlorophyll content was 1.94 mg/g FW, significantly higher than that of control leaves (**Figure 6D**). When the FM concentration was increased to 20%, the anti-aging effect increased significantly, and the chlorophyll content increased to 2.51 mg/g FW. Further increasing of FM concentration did not significantly increase the chlorophyll content further. Taken together, the anti-aging strain ITBB2-31 screened by *W. microscopica*-ammonia system is effective to other plants.

### 3.6 Comparative metabolome analysis reveals both anti-aging and growth-inhibiting agents in the fermented medium of ITBB2-31

**Figure 7:**
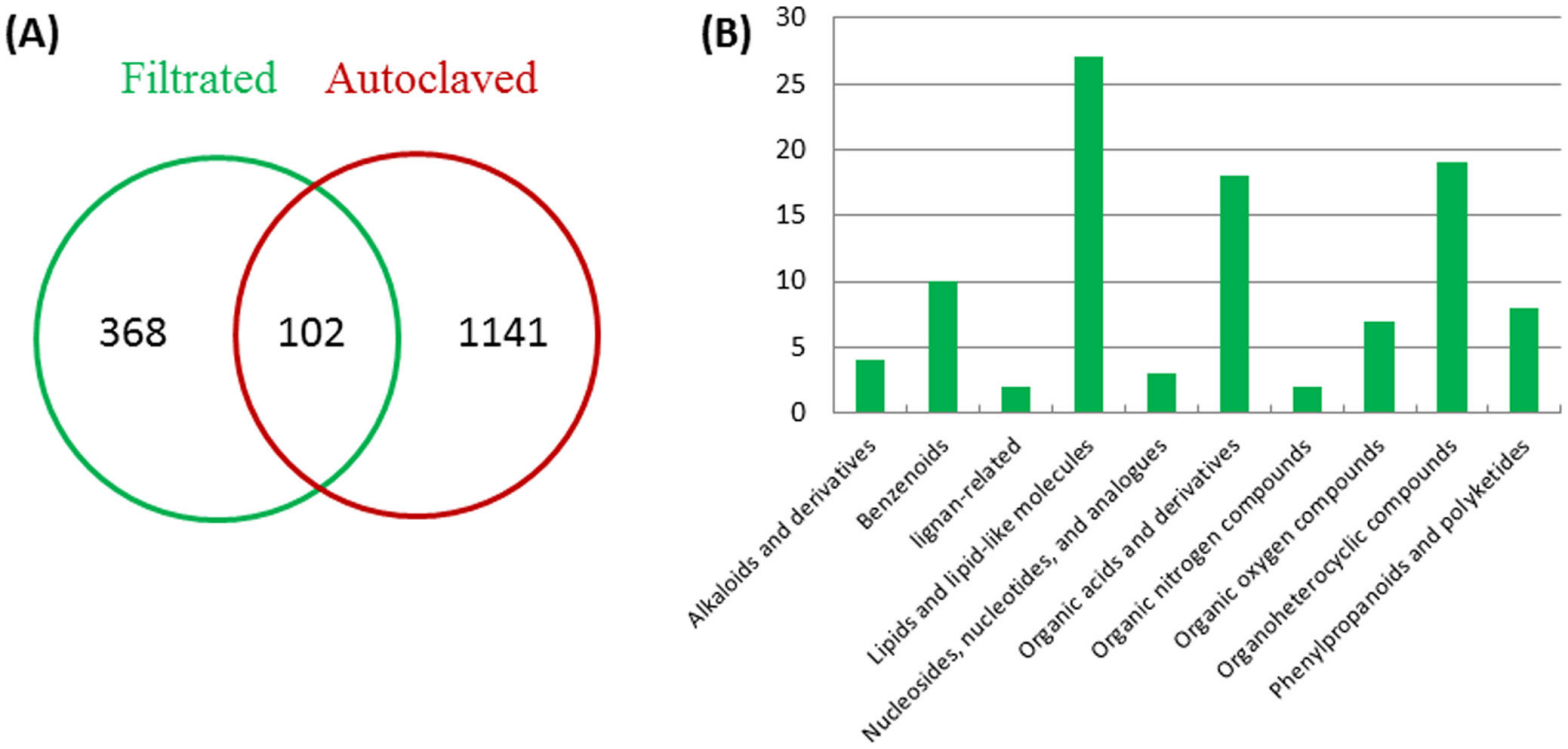
Classification of metabolites in the filter-sterilized and autoclaved FM. **(A)** numbers of shared and distinct compounds in the filter-sterilized and autoclaved FM; **(B)** classification of compounds shared by two media.

LC-ESI-MS/MS identified 470 compounds in the filter-sterilized FM (**Supplementary Table S2**), among which 368 compounds disappeared after autoclaving (**Figure 7A**), while 1141 new compounds that were supposed to be driven from heat-degradation were identified in the autoclaved FM (**Figure 7A**; **Supplementary Table S3**). The anti-aging agents are heat-stable (**Figure 4**) and are supposed to present in both filter-sterilized and autoclaved FMs with similar abundances. In total, 102 compounds are shared by both treatments (**Figure 7A**; **Supplementary Table S4**). They are classified into ten subgroups, including lipids and lipid-like molecules (Fu et al., 2017), organic acids and derivatives (Appenroth, 2015), Organoheterocyclic compounds (Sree and Appenroth, 2016), Benzenoids (Zhang et al., 2010), and others (Zheng et al., 2009c) (**Figure 7B**). Seventeen compounds have similar relative abundances in both treatments (0.75 < Rf/Ra < 1.5, Rf (Relative abundance of Filter-sterilized FM), Ra (Relative abundance of Autoclaved FM), **Supplementary Table S4**) and are potentially the anti-aging candidates, out of which, some have been reported to have anti-aging effects in animals and/or plants, including 3-(2,4-dihydroxypentyl)-8-hydroxy-6-methoxyisochromen-1-one (an isocoumarin derivative) (Powers et al., 1989), indole-3-acetaldehyde (McClerklin et al., 2018), leupeptin (Carlin et al., 1994), muramic acid (Lehtonen et al., 1997), alpha-lapachone (Cho et al., 2006), and fatty acyls (Yu et al., 2020). Therefore, the anti-aging effects may come from a combination of compounds.

Compounds that inhibited the growth of *W. microscopica* are sensitive to heat-treatment and presented weak inhibition effect in the autoclaved FM (**Figure 4**), therefore, the growth-inhibiting agents are supposed to have significantly reduced abundance in the autoclaved FM. We set a cutoff value of 90% reduction of relative abundance in the autoclaved FM, which is equivalent to Rf/Ra > 9. Among the compounds that are shared in both treatments, 13 compounds mainly organic acids, alkaloid derivatives, and nucleoside analogues, have a reduction of > 90% in the autoclaved FM (**Supplementary Table S4**), some of which have been reported to have toxic and/or growth-inhibiting effects, including S-adenosyl-L-homocysteine (Penyalver et al., 2009), 4-hydroxyquinoline (Chung et al., 1989; Inderjit, Bajpai and Rajeswari, 2010), ginkgolide A (Weakley et al., 2011), phenylalanine (Nguyen et al., 2014; Cao et al., 2019), sparteine-15-carboxylic acid (Ashcroft et al., 1991), 3-hydroxypropanoic acid (Yung et al., 2016), and 2-hydroxyglutaric acid (Bjugstad et al., 2001; Keyser et al., 2008). Therefore, the growth-inhibiting effect of strain ITBB2-31 may come from a combination of compounds, similar to the anti-aging effect.

## 4 Discussion

### 4.1 *W. microscopica* is the duckweed species most sensitive to ammonium-induced senescence

*W. microscopia* is distributed in the Indian subcontinent. It was first named *Grantia microscopica* (Griff. Ex Voigt) (Voigt, 1845; Griffith, 1851), and was later identified as a member of the genus Wolffia Horkel (Kurz, 1866). Many investigations have been carried out on this plant since its identification (Hegelmaier, 1885; Maheshwari SC and Chauhan OS, 1963; Maheshwari SC and Venkataraman, 1966; Roy and Dutt, 1967; Bakshi et al., 1979; Khurana et al., 1986; Landolt, 1986). However, this species could have been causing trouble since its discovery (Hegelmaier, 1885), and could not be found for decades at places where it was discovered (Sree et al., 2015). The axenic clones of *W. microscopica* were once lost completely from all duckweed collections in the year 2009 (Sree and Appenroth, 2014), however, rediscovered from the lakes at Patan, Ambapur, Sughad and Vadasma in Gujarat, India and from that at Jessore, Bangladesh in 2013 (Sree and Appenroth, 2014). We obtained two of the newly collected clones 2001 and 2008 from K-J Appenroth in 2014, and immediately realized that they were more difficult to maintain than the other duckweed species.

In this research, we tested four commonly used duckweed culture media and found that MH and SH were not good media to keep *W. microscopia* alive for as long time as the other duckweed species. *W. microscopica* clones 2001 and 2008 performed similarly on these media and grew rapidly in the beginning, and then stopped growth and died within one month (**Figure 2**). In contrast, the other species can be subcultured at three-month intervals without problem. By comparing the components in the four media, we found that both MH and SH media contained ammonium ion, while E and N media that did not facilitate early senescence did not contain ammonium ion. Therefore, ammonia was supposed to be the inducer for the early-senescence in *W. microscopica*. This hypothesis was further confirmed to be right by supplementing ammonia in the E and N media that do not contain ammonium ion (**Figure 2C**). A concentration of 0.5 mM of ammonia was enough to induce early senescence in both strains of *W. microscopica* (**Figures 2D, E**).

### 4.2 The high sensitivity of *W. microscopica* to ammonia-induced senescence makes it a useful tool to screen for plant anti-aging microorganisms

There have been many reports on plant growth-promoting effects of endophytic microorganisms, which has been well reviewed (Aly et al., 2011; Ma et al., 2011; Santoyo et al., 2016). These endophytes are highly diverged and include both fungi (Harzallah et al., 2012; Larriba et al., 2015; Khan et al., 2017) and bacteria (Shi et al., 2010; Paz et al., 2012; Quecine et al., 2012; You et al., 2012). Some endophytic strains previously isolated from the rubber tree have been shown to have antifungal activities (Zheng et al., 2009a), and have a potential in biological control of plant pathogenic diseases (Tan et al., 2015).

In this research, we used *W. microscopica* to screen the endophytic fungal strains isolated from rubber tree (Zheng et al., 2009b; Tan et al., 2015) for plant anti-aging microorganisms, and found one of the strains, ITBB2-31 that strongly postponed aging and promoted the growth of *W. microscopica* (**Figures 2**-**5**). The stay-green period of the axenic stock cultures was extended from less than one month to at least seven months (**Figure 5**). This strain also extended the lifespan of the other duckweed strains in the genus Wolffia. Interestingly, besides the anti-aging and growth promoting effects, the fermented broth of ITBB2-31 inhibited the growth of *W. microscopica* at high concentrations, which was detoxified by autoclaving (**Figure 4**), suggesting that ITBB2-31 has both promotion and inhibition effects on plant growth.

### 4.3 Anti-aging activity of microorganisms sreened by the *W. microscopica-*Ammonia system is effective to inhibit senescence of the other plant species

The anti-aging microorganism ITBB2-31 was screened using the *W. microscopica-*ammonia system. To test whether it is effective to inhibit the senescence of other plant species, we used a dark-detached system to induce senescence of the leaves of rubber tree and cassava. Darkness is an extreme light condition and is often used to induce rapid and synchronous senescence in detached leaves (Weaver and Amasino, 2001), and the dark-detached system has been widely used as a senescence model to study the age-triggered senescence (Song et al., 2014; Wei et al., 2017). Our results indicated that the exudates of ITBB2-31 was effective to inhibit dark-induced senescence of detached leaves of rubber tree and cassava (**Figure 6**), suggesting that ammonia-induced senescence and dark-induced senescence may have shared signal transduction pathway.

### 4.4 Potential anti-aging and growth-inhibiting agents in ITBB2-31

Multiple compounds with either anti-aging or growth-inhibiting activities were identified through comparative metabolome analysis of filter-sterilized and autoclaved media of strain ITBB2-31. Both activities are suggested to come from a combination of compounds, since multiple compounds in the metabolites have been reported to have anti-aging or growth-inhibiting activities. In this research, a total of 1188 compounds were identified by LC-ESI-MS/MS analysis, and 102 compounds were shared by both filter-sterilized and autoclaved FMs (**Figure 7A**; **Supplementary Table S4**). To narrow down our search for the anti-aging compounds, we supposed the anti-aging agents to have similar relative abundance in both treatments, and set a cutoff value of 0.75 < Rf/Ra < 1.5, since the anti-aging agents are heat-stable (**Figure 4**). Seventeen compounds lay in the search scope, and biological functions of them were mined in literatures. Results indicated that some of them have been reported to have anti-aging effects in animals and/or plants, including 3-(2,4-dihydroxypentyl)-8-hydroxy-6-methoxyisochromen-1-one (an isocoumarin derivative) (Powers et al., 1989), indole-3-acetaldehyde (McClerklin et al., 2018), leupeptin (Carlin et al., 1994), muramic acid (Lehtonen et al., 1997), alpha-lapachone (Cho et al., 2006), and fatty acyls (Yu et al., 2020). Therefore, the anti-aging effects may come from a combination of compounds. Isocoumarin and derivatives have been shown to inhibit the activity of a variety of serine proteases (Powers et al., 1989), and potentially inhibit senescence. Indole-3-acetaldehyde is a precursor of indole-3-acetic acid (IAA) in Arabidopsis and is converted to IAA through a novel pathway (McClerklin et al., 2018). Leupeptin specifically inhibit the proteolytic activity in senescent cell lysates and potentially inhibit senescence of plants (Carlin et al., 1994). Muramic acid interacts with lectin in plants (Ayouba et al., 1991), and were detected in more percentage in young people than in the olds (Lehtonen et al., 1997). A plant alpha-lapachone has been proven to have antivascular activity in animal cells (Garkavtsev et al., 2011) and antifungal activity in plants (Cho et al., 2006). Benzoic acid derivatives from *Bjerkandera adusta* has been shown to modulate the proteostasis network, and likely to be anti-aging agents (Georgousaki et al., 2020). Fatty acyls are potential biomarkers related to the anti-aging effect of ginsenoside Rb1 (GRb1), an active ingredient of traditional Chinese medicine *Panax ginseng* C. A. Meyer (Yu et al., 2020).

To narrow down our search for potential growth-inhibiting agents, we set a cutoff value of 90% reduction of relative abundance in the autoclaved FM compared to that in the filter-sterilized FM, which is equivalent to Rf/Ra > 9, since compounds that inhibited the growth of *W. microscopica* are sensitive to heat-treatment and presented only weak inhibition effect in the autoclaved FM (**Figure 4**). Among the compounds shared in both treatments, 13 compounds lay in our search scope, some of which have been reported to have toxic and/or growth-inhibiting effects, including S-adenosyl-L-homocysteine (Penyalver et al., 2009), 4-Hydroxyquinoline (Chung et al., 1989; Inderjit, Bajpai and Rajeswari, 2010), Ginkgolide A (Weakley et al., 2011), Phenylalanine (Nguyen et al., 2014; Cao et al., 2019), sparteine-15-carboxylic acid (Ashcroft et al., 1991), 3-hydroxypropanoic acid (Yung et al., 2016), and 2-hydroxyglutaric acid (Bjugstad et al., 2001; Keyser et al., 2008). S-adenosyl-L-homocysteine is a nucleotide analogue, its hydrolase gene ahcY is required for optimal growth of *Agrobacterium radiobacter* K84 (Penyalver et al., 2009), suggesting that S-adenosyl-L-homocysteine is a growth inhibitor. In our research, S-Adenosyl-L-homocysteine has a high content before autoclaving, but only 4% left after autoclaving, suggesting that it may take part in the growth inhibition of *W. microscopica*. 4-Hydroxyquinoline is an inhibitor of NADH-ubiquinone reductase in the respiratory chain of mitochondria (Chung et al., 1989); a similar compound, 8-hydroxyquinoline (HQ) is exuded from the roots of *Centaurea diffusa*, and reduce growth of the other plants (Inderjit, Bajpai and Rajeswari, 2010). Ginkgolide A inhibits vascular smooth muscle proliferation and reduces neointimal hyperplasia in a mouse model (Weakley et al., 2011). Phenylalanine inactivates kinases (Nguyen et al., 2014) and intestinal digestive enzymes (Cao et al., 2019) activities, and thus inhibits development. Sparteine derivatives are well known toxic alkaloids (Pothier et al., 1998), and have been shown to inhibit growth of monocots (Parmaki et al., 2018). 3-hydroxypropanoic acid has been shown to inhibit the growth of *Escherichia coli* (Yung et al., 2016).

Besides the compounds mentioned above, some other metabolites in the fermented media may have also taken part in either the anti-aging or the growth-inhibiting activities, since the cutoff values we used in the comparative analysis were relatively arbitrary to narrow down the search, however, they contribute only to a larger combination of the compounds.

### 4.5 Possible mechanism of ammonia-induced senescence in plants

Ammonium is often the preferred form of N nutrition for some higher plants such as eucalyptus (Pfautsch et al., 2009) and rice (Song et al., 2013), and to some extent delays leaf aging (Diaz et al., 2008; Aguera et al., 2010). However, ammonium inhibits the growth of many other plant species, including *Arabidopsis thaliana* (Podgorska et al., 2017). The toxicity of ammonium is associated with changes in the cellular redox state (Podgorska et al., 2015), and uncoupling of electron transport and respiration inhibition (Hachiya and Noguchi, 2011). The duckweed species *W. microscopica* is extremely sensitive to ammonium toxicity. In this study, a concentration of 0.5 mM of ammonium was enough to inhibit the growth of *W. microscopica* and induce its senescence. Moreover, the ammonium-induced senescence seemed to be associated with dark-induced senescence. The exudates of fungal strain ITBB2-31 that inhibited ammonia-induced senescence, inhibited dark-induced senescence of the rubber tree and cassava leaves (**Figure 6**). Therefore, ammonia- and dark-induced senescence must have shared their signal transduction pathway. Ammonia-induced senescence has been reported in both mammals and plants (Chen and Kao, 1996; Gorg et al., 2015). Dark-induced senescence of detached maize leaves and water-stress induced senescence of detached rice leaves were associated with accumulation of ammonium ion (Chen and Kao, 1996; Lin and Kao, 1998). A decrease of glutamine synthase GS activity attributed to ammonium accumulation in maize and rice leaves (Chen and Kao, 1996; Lin and Kao, 1998; Chien and Kao, 2000), which in turn, increased tissue sensitivity to ethylene and accelerated leaf senescence (Lin and Kao, 1998). However, a later report showed that ammonia accumulation in detached rice leaves did not change tissue sensitivity to ethylene, and ruled out the possible involvement of ethylene in ammonia-induced senescence (Lin et al., 2002). Calcium effectively reduced ammonium-promoted senescence of detached rice leaves, suggesting that ammonium-induced senescence may be mediated through blocking the entrance of calcium ions into the cytosol (Hung and Kao, 1998).

## 5 Conclusions

*W. microscopica* was showed to be highly sensitive to ammonia-induced senescence, and can be served as a model plant to screen for plant anti-aging microorganisms. By co-culturing *W. microscopica* with endophytic microorgainisms isolated from rubber tree, an *Aspergillus sclerotiorum* strain ITBB2-31 that dramatically increased the lifespan and the biomass of *W. microscopica* was selected. Interestingly, both filter-sterilized and autoclaved exudates of this fungal strain prolonged the lifespan of *W. microscopica* cultures from 1 month to at least 7 months. Moreover, the anti-aging effect of this fungal exudate on the rubber tree and cassava leaves was significant, with an increase of chlorophyll contents by 50% - 350%. Additionally, high contents of filter-sterilized exudates remarkably restrained the growth of *W. microscopica* while extending its lifespan, but high contents of autoclaved exudates presented a weak inhibition effect of its growth. These results indicated that there were both heat-sensitive growth-inhibiting and heat-stable anti-aging agents in the exudates. Comparative metabolome analysis of the filter-sterilized and autoclaved exudates revealed multiple heat-stable anti-aging and heat-sensitive growth-inhibiting compounds. Our findings will be useful in large scale screening of microbes and compounds with anti-aging function, and will be beneficial to agriculture.

## Data Availability

The datasets presented in this study can be found in online repositories. The names of the repository/repositories and accession number(s) can be found in the article/**Supplementary Material.**
