# Supplementary material for: Screening method and metabolic analysis of plant anti-aging microorganisms via ammonia-induced senescence in the duckweed Wolffia microscopica
Source: Front Plant Sci. 2024 Nov 13;15:1480588. doi: 10.3389/fpls.2024.1480588 (PMC11605829; doi:10.3389/fpls.2024.1480588)
Supplement: Supplementary file 1 [file Table1.docx]

Table S1. Basal salts in the media used in this study

| Chemicals | SH (µM) | MH (µM) | E (µM) | N (µM) |
| --- | --- | --- | --- | --- |
| NH_4_NO_3_ |  | 1000 |  |  |
| KNO_3_ | 12400 | 5000 | 15000 | 8000 |
| KH_2_PO_4_ |  | 1000 | 5000 | 150 |
| NH_4_H_2_PO_4_ | 1300 |  |  |  |
| MgSO_4_·7H_2_O | 800 | 2000 | 2000 | 1000 |
| Ca(NO_3_)_2_·4H_2_O |  | 4000 | 5000 | 1000 |
| CaCl_2_·2H_2_O | 680 |  |  |  |
| H_3_BO_3_ | 40 | 100 | 46 | 5 |
| MnCl_2_·4H_2_O |  |  | 18 | 13 |
| MnSO_4_·H_2_O | 30 | 100 |  |  |
| ZnSO_4_·7H_2_O | 1.74 | 30 | 0.8 |  |
| CuSO_4_·5H_2_O | 0.4 | 0.1 | 0.3 |  |
| H_2_MoSO_4_·H_2_O |  |  |  |  |
| Na_2_MoSO_4_·2H_2_O | 0.2 | 1 | 0.5 | 0.4 |
| KI | 3 | 5 |  |  |
| CoCl_2_·6H_2_O | 0.21 | 0.1 |  |  |
| FeSO_4_·7H_2_O |  | 100 |  |  |
| FeCl_3_·6H_2_O | 260 |  | 20 | 25 |
| Na_2_EDTA | 260 | 100 |  | 25 |
| EDTA |  |  | 30 |  |
| tartaric acid |  |  | 20 |  |
| pH |  | 5.7 | 4.6 | 5.5 |

Note. MH—Modified Hoagland medium (Hoagland and Arnon 1950)

E—E-medium (Cleland and Briggs 1967).

N medium (Appenroth et al. 1996).

SH medium (Schenk and Hildebrandt 1972).

*References:*

Appenroth K-J, Teller S, Horn M (1996) Photophysiology of turion formation and germination in *Spirodela polyrhiza*. Biologia Plantarum 38:95-106

Cleland CF, Briggs WR (1967) Flowering responses of the long-day plant *Lemna gibba* G3. Plant physiology 42 (11):1553-1561

Hoagland DR, Arnon DI (1950) The water-culture method for growing plants without soil. California Agricultural Experiment Station Circular 347:1-32

Schenk RU, Hildebrandt AC (1972) Medium and techniques for induction and growth of monocotyledonous and dicotyledonous plant cell cultures. Can J Bot 50:199-204
