# Supplementary figures and images for: Screening method and metabolic analysis of plant anti-aging microorganisms via ammonia-induced senescence in the duckweed Wolffia microscopica
Source: Front Plant Sci. 2024 Nov 13;15:1480588. doi: 10.3389/fpls.2024.1480588 (PMC11605829; doi:10.3389/fpls.2024.1480588)

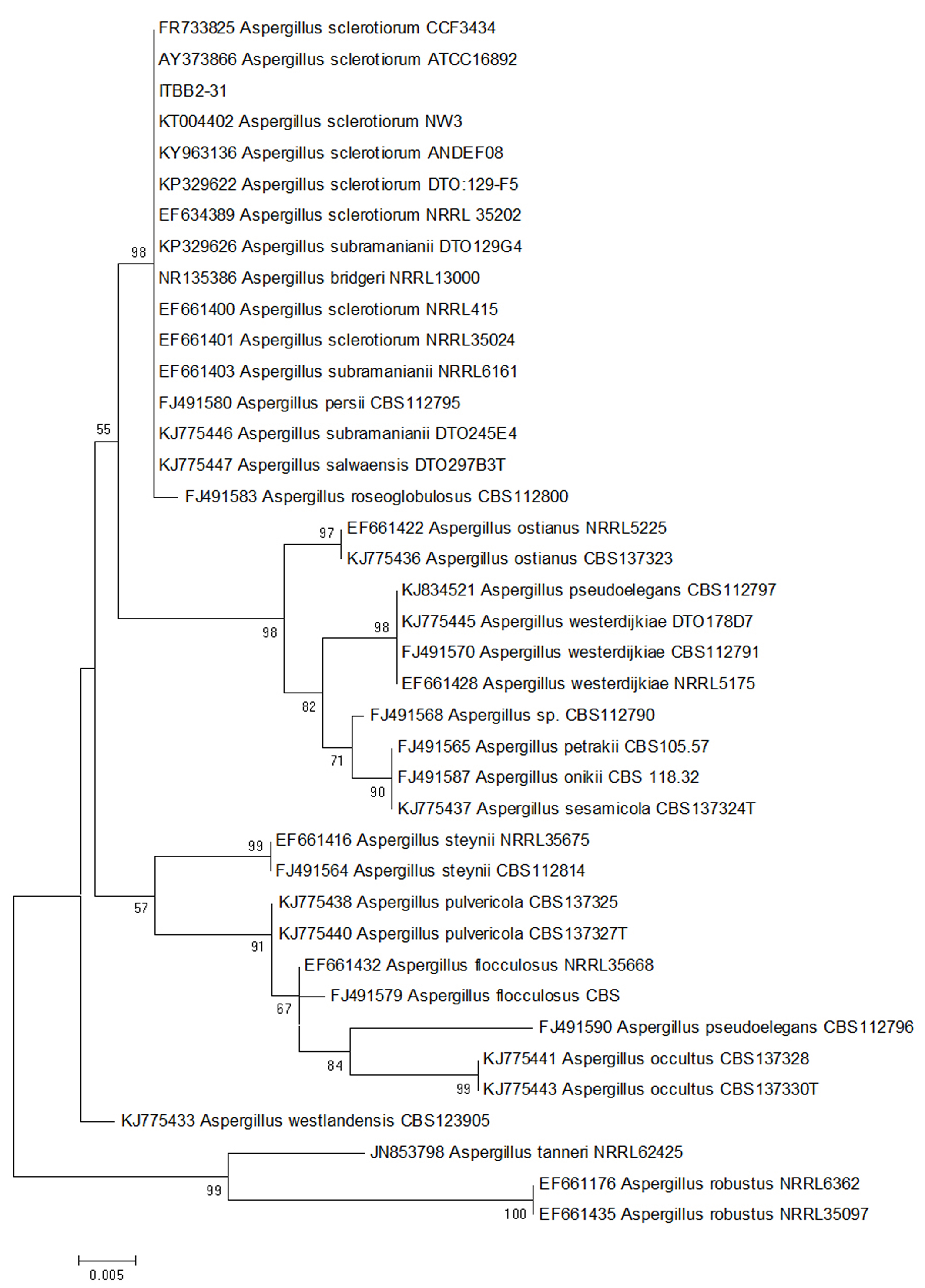

Supplement: Supplementary file 3 [file Image1.jpeg]
